# Heat-killed *Limosilactobacillus reuteri* PSC102 Ameliorates Impaired Immunity in Cyclophosphamide-induced Immunosuppressed Mice

**DOI:** 10.3389/fmicb.2022.820838

**Published:** 2022-08-12

**Authors:** Md. Sekendar Ali, Eon-Bee Lee, Yixian Quah, Biruk Tesfaye Birhanu, Kyoungho Suk, Suk-Kyung Lim, Seung-Chun Park

**Affiliations:** ^1^Department of Biomedical Science and Department of Pharmacology, School of Medicine, Brain Science and Engineering Institute, Kyungpook National University, Daegu, South Korea; ^2^Laboratory of Veterinary Pharmacokinetics and Pharmacodynamics, College of Veterinary Medicine, Kyungpook National University, Daegu, South Korea; ^3^Department of Pharmacy, International Islamic University Chittagong, Kumira, Bangladesh; ^4^Cardiovascular Research Institute, Kyungpook National University, Daegu, South Korea; ^5^Bacterial Disease Division, Animal and Plant Quarantine Agency, Gimcheon-si, South Korea

**Keywords:** *Limosilactobacillus reuteri* PSC102, cyclophosphamide, lymphocytes, cytokines, immune stimulation

## Abstract

The immune functions of heat-killed *Limosilactobacillus reuteri* PSC102 (hLR) were investigated in cyclophosphamide (CP)-treated immunosuppressed mice. BALB/c mice were randomly divided into five groups: normal control group, CP group, CP treated with levamisole (positive control group), and CP treated with low- and high-dose hLR. After receiving the samples for 21 days, mice were sacrificed, and different parameters, such as immune organ index, immune blood cells, splenocyte proliferation, lymphocyte subpopulations, cytokines, and immunoglobulins, were analyzed. Results showed that the immune organ (thymus and spleen) indices of hLR treatment groups were significantly increased compared to the CP group (*p* < 0.05). hLR administration prevented CP-induced reduction in the numbers of white blood cells, lymphocytes, midrange absolute, and granulocytes, providing supporting evidence for hematopoietic activities. Splenocyte proliferation and T-lymphocyte (CD4^+^ and CD8^+^) subpopulations were also significantly augmented in mice treated with hLR compared to the CP group (*p* < 0.05). Moreover, Th1-type [interferon-γ, interleukin (IL)-2, and tumor necrosis factor-α] and Th2-type (IL-4 and IL-10) immune factors and immunoglobulin (IgG) showed significant increasing trends (*p* < 0.05). Additionally, the other proinflammatory cytokines (IL-1β and IL-6) were also significantly elevated (*p* < 0.05). Taken together, this investigation suggested that orally administered hLR could recover immunosuppression caused by CP and be considered a potential immunostimulatory agent for the treatment of immunosuppressive disorders.

## Introduction

Immunosuppression is a state of immune dysfunction that decreases the immune response to antigens and makes the individual more sensitive to diseases (Round and Mazmanian, 2009). Based on the condition of immunosuppression, patients may be at risk for different kinds of infections and complications, delaying diagnostic and treatment outcomes. For example, the immunocompromised conditions of HIV infection, irritable bowel disease, and cancer often lead to low antibody levels or ineffective treatment (McGrath et al., 2020). To control viral infectious diseases and prevent infections, either vaccines or immunostimulating drugs at a high dose need to be used for long-term treatment, often leading to serious side effects (Xie et al., 2015). Hence, the most effective way of treating and preventing immunosuppressive disorders is to explore and develop novel immunostimulators.

Nowadays, the application of probiotics has acquired considerable attention as a treatment option for immune diseases. Probiotics play an important role in maintaining the intestinal microbiota balance, immunomodulation, preventing gastrointestinal infections as well as improving hematological indices (Park et al., 2017). Previous studies have shown that lactobacilli can be effective for host immunostimulation to build the early line of defenses against pathogenic infections (Bujalance et al., 2007; Paturi et al., 2007). *Limosilactobacillus* (formerly *Lactobacillus*) *reuteri* (*L. reuteri*) (Zheng et al., 2020) has been reported to show immunoregulatory activity by the activation of macrophage populations, IgA and IgG production (De Gregorio et al., 2016), prevention of streptococcus infections (De Gregorio et al., 2015), promotion of Th-cell responses (Hoang et al., 2019), production of biogenic amines (Greifová et al., 2017), and enhancement of the gut cytokine profile (Maassen et al., 2000). Moreover, *L. reuteri* has been proven effective for treating infantile colic (Sung et al., 2018) and gastrointestinal (Francavilla et al., 2008) infections.

However, the safety issue of using live probiotics is still a matter of argument. The potential risks could be systemic infections due to translocation in pediatric and vulnerable patients (Deshpande et al., 2018). Additionally, probiotic administration may cause the risk of acquiring antibiotic resistance genes and intervening in the gut microbiota in neonates (Bafeta et al., 2018). The use of live probiotics in certain risk factors, such as diabetes mellitus, mitral regurgitation, and short-gut syndrome, may produce different forms of sepsis, including liver abscess, endocarditis, and bacteremia (Mackay et al., 1999; Rautio et al., 1999; Kunz et al., 2004). To overcome these risks, there is a growing interest in using non-viable probiotic bacteria or their cell extracts, mainly heat-inactivated lactic acid probiotic bacteria. Heat-killed probiotic cells, cell-free supernatants, and isolated, purified main components can produce beneficial effects, including immunomodulation (Kataria et al., 2009; Taverniti and Guglielmetti, 2011). Heat-killed *Limosilactobacillus rhamnosus* OLL2838 has been shown to protect the mucosal barrier integrity defects in colitis-induced mice (Miyauchi et al., 2009). Heat-killed *Limosilactobacillus paracasei* can boost immunity by stimulating splenocyte and macrophage proliferation (Chiang et al., 2012). The combined therapy of several heat-killed probiotics can modulate the production of cytokines and immunoglobulins (Campeotto et al., 2011; Chang et al., 2013). Moreover, heat-killed probiotics may also provide the development of safer preparations with pharmaceutical properties, such as reduction of reactions with other materials, easier storage, and extension of shelf-life (Kang et al., 2021). In this study, *L. reuteri* PSC102, a newly identified probiotic strain isolated from pigs, is yet to check for potential immunoregulatory effects. Therefore, this study aimed to investigate the immunostimulatory effects of heat-killed *L. reuteri* PSC102 (hLR) in cyclophosphamide (CP)-induced immunosuppressed BALB/c mice. CP is a classical antineoplastic drug that causes immunosuppression, oxidative stress, and physiological disturbance as side effects and is widely used to produce an immunosuppressive model (Xie et al., 2016; Meng et al., 2019).

## Materials and Methods

### Chemicals and Reagents

Cyclophosphamide monohydrate and levamisole hydrochloride were purchased from Tokyo Chemical Industry Co., Ltd. (Tokyo, Japan). 3-(4,5-Dimethylthiazol-2-yl)-2,5-diphenyltetrazolium bromide (MTT), concanavalin A (Con A), lipopolysaccharide (LPS), and Roswell Park Memorial Institute (RPMI)-1640 medium were obtained from Sigma-Aldrich (St. Louis, MO, United States). Cytokines and IgG enzyme-linked immunosorbent assay (ELISA) kits were purchased from ELK Biotechnology (Wuhan, China). Red blood cell (RBC) lysing buffer, fetal bovine serum (FBS), and penicillin-streptomycin (P/S) were purchased from Gibco Life Technologies (New York, NY, United States). Dimethyl sulfoxide (DMSO) was obtained from Duksan Pure Chemical Co., Ltd. (Kyungkido, South Korea). Phosphate-buffered saline (PBS) was provided by Welgene, Inc. (Gyeongsangbuk, South Korea). Anti-CD4^+^ [phycoerythrin (PE)] and anti-CD8^+^ [fluorescein isothiocyanate (FITC)] antibodies were supplied by BD Biosciences (San Diego, CA, United States).

### Isolation and Preparation of Sample

Gram-positive probiotic *Limosilactobacillus reuteri* PSC102 (*L. reuteri* PSC102) is a patent strain isolated from pig feces (*Sus domesticus*). The analysis details have been deposited in the repository,^1^ providing the GenBank accession code (MZ127631.1). The strain also has accession number given by the International depository authority (KCCM12927P) as a patent strain. Briefly, feces were obtained in sterile plastic bags from Gyeongsangbuk Veterinary Service Laboratory (Daegu, South Korea). Fecal samples were mixed with buffered peptone water broth, followed by shaking for 2 min. The mixed samples were spread on a de Man Rogosa and Sharpe (MRS) agar plate and incubated for 24 h at 37°C. The colonies were randomly selected from plates and individually cultivated in MRS broth at 37°C for 24 h and restreaked onto the MRS agar. The isolates were subjected to Gram staining and microscopic observation.

To prepare samples for *in vivo* experiments, *L. reuteri* PSC102 was grown in MRS medium for 24 h at 37°C. The pellets were collected by centrifugation for 10 min at 6,000 rpm at 4°C, and the culture supernatant was discarded. The cell pellets were washed twice with sterile PBS. The collected pellets were dried by lyophilization in a vacuum freeze dryer (Operon Co., Ltd, Gyeonggi, South Korea). The colony-forming units per gram (CFU/g) of the dried sample was determined by serial 10-fold dilution and incubation on an MRS agar plate for 24 h at 37°C. The dried pellet was heat-killed at 80°C for 15 min in a temperature-controlled water bath. Non-viability was confirmed by plating the inactivated sample on MRS agar medium and incubating overnight at 37°C. There were no bacterial colonies observed.

### Scanning Electron Microscopy Analysis

Scanning electron microscopy (SEM) was used to examine the morphological characteristics of hLR, as described previously (Vinod et al., 2015). Heat-inactivated and non-treated control *L. reuteri* PSC102 cells were fixed for 2 h at 4°C in 2.5% glutaraldehyde in PBS (pH 7.0) and washed thrice with the same buffer. Cells were dehydrated in a graded sequence of ethanol concentrations (30, 50, 70, 80, 90, and 100%) after washing. The samples were kept overnight at –70°C and dried by lyophilization for 24 h. The prepared samples were mounted on an SEM tube and sputter-coated with gold-palladium before analysis on an SEM (model S-4300; Hitachi, Tokyo, Japan) at 5.0 kV voltage and ×20,000 magnification.

### 16S rRNA Gene Sequencing Analysis

The genomic DNA of PSC102 was isolated using the DNA isolation and purification kit (Qiagen, Hilden, Germany) according to the manufacturer’s protocol. The universal primer (27F/1492R) was applied, and the following cycling conditions were maintained for analysis: enzyme activation and initial denaturation at 94°C for 5 min, 35 cycles of denaturation for 30 s at 94°C, annealing for 30 s at 56°C, and elongation for 30 s at 72°C, followed by final extension for 7 min at 72°C. The polymerase chain reaction product was analyzed from the Korean Culture Center of Microorganisms (Seoul, South Korea). Using BLAST software, the sequencing results were compared to closely correlated sequences available from the GenBank database. Finally, a phylogenetic tree was constructed by the neighbor-joining method using MEGA 5.0 software (An et al., 2006).

### Experimental Animals

Forty specific pathogen-free male BALB/c mice (4–5 weeks, 18 ± 20 g) were purchased from Central Lab Animal, Inc. (Seoul, South Korea). All mice were acclimatized to rodent facilities for 1 week before the commencement of the experiment. Mice were provided standard laboratory conditions at 24 ± 1°C, 55 ± 5% relative humidity, and 12/12 light/dark cycle and fed standard laboratory chow feed and water *ad libitum*. All experimental protocols were approved by the Laboratory Animal Care and Use Committee of Kyungpook National University (2020-103).

### Experimental Design

**FIGURE 1:**
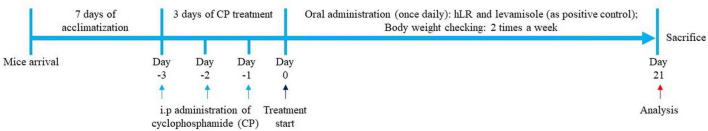
Experimental timeline.

After acclimatization to the laboratory environment, all mice were randomly divided into five groups consisting of eight mice each (Table 1). The total number of mice used in the study was calculated by the G*power program (3.1.9.2) based on effect size (0.5), α error probability (0.05), power (1-β error probability) (0.8), and group number (5). Except for the normal control (NRM) group, mice from the other four groups were intraperitoneally (i.p.) injected with CP (80 mg/kg body weight/day) in a sterile isotonic saline solution for the first three consecutive days to induce immunosuppression (CP group). Oral administration was conducted with 200 μL levamisole (80 mg/kg; PC group), low-dose hLR (hLR-L; 10^6^ CFU/kg), and high-dose hLR (hLR-H; 10^10^ CFU/kg) once daily for 21 days (Figure 1). The samples were dissolved in distilled water and administered into mice through gavage feeding. The body weight was checked twice weekly to adjust the dose. After 21 days, all mice were sacrificed, and blood and immune organs were processed accordingly.

**TABLE 1:** Description of different groups.

Groups	Treatment	Description
NRM	Normal control	Normal diet and water
CP	CP control	i.p. administration with three consecutive doses of 80 mg/kg CP
PC	CP and positive control	i.p. administration of three consecutive doses of 80 mg/kg CP and treated with 40 mg/kg levamisole
hLR-L	CP and low-dose treatment	i.p. administration of three consecutive doses of 80 mg/kg CP and treated with 1 × 10^6^ CFU/kg/day hLR
hLR-H	CP and high-dose treatment	i.p. administration of three consecutive doses of 80 mg/kg CP and treated with 1 × 10^10^ CFU/kg/day hLR

#### Body Weight Analysis

The body weight of each mouse of the five groups was measured at 3-day intervals for a total of nine times throughout the experiments.

#### Determination of Immune Organ Index

On day 21, after treatments, all mice in each group were terminated by cervical dislocation, and the thymus and spleen were removed and weighed immediately. Finally, the organ indices were calculated as follows:


$$\begin{array}{l} \text{Index =}\frac{\text{weight of thymus or spleen }\left(\text{mg}\right)}{\text{body weight }\left(g\right)}\left(Qietal.\text{, 2018}\right) \end{array}$$

#### Determination of Hematological Parameters

After hLR administration for 3 weeks, blood was collected from the tail vein of mice and kept in EDTA-coated Microvette^®^ CB 300 K2E tubes (Sarstedt AG and Co., Numbrecht, Germany). The number of white blood cells (WBCs), lymphocytes, midrange absolute (MID), and granulocytes in each blood sample was measured using URIT-300 Vet Plus (URIT Medical Electronic Co., Ltd., Guangxi, China).

#### Preparation of Thymocytes and Splenocytes

Thymocyte and splenocyte isolation and preparation were done according to a previously described method (Han et al., 2018). Aseptically collected thymus and spleen from mice were gently passed through a 70 μm cell strainer (BD Biosciences) using the plunger of a 3 mL syringe. Cells were collected and washed thrice with RPMI-1640 by centrifugation (1800 rpm, 5 min, 4°C). The collected cells were treated with RBC lysing buffer, and lysed RBCs were removed by centrifugation and maintained in RPMI-1640 supplemented with 10% FBS and 1% P/S.

#### Mitogen Con A- and LPS-induced Splenocyte Proliferation Assay

The proliferation of splenocytes was determined using the MTT assay (Zhang et al., 2020). The number of splenocytes was adjusted to 1 × 10^6^ cells/well in RPMI-1640 complete medium and seeded at 100 μL/well in a 96-well plate. Next, mitogen Con A and LPS (5 μg/mL) were added to each well to make the final volume of 200 μL, followed by incubation for 72 h at 37°C. The wells containing only RPMI-1640 medium without any mitogen were used as control. The MTT (5 mg/mL) solution (20 μL) was added to each well, followed by incubation for another 4 h. The medium was removed, and 200 μL DMSO was added to each well and shaken for 10 min in an orbital shaker. Finally, absorbance was measured at 570 nm using Gen5 microplate reader version 3.08 (BioTek, Winooski, VT, United States).

#### Flow Cytometric Analysis of Thymic and Splenic T-lymphocyte Subpopulations

The above-mentioned prepared thymic and splenic lymphocytes were adjusted to 1 × 10^6^ cells/mL, mixed with anti-CD3^+^ (APC), anti-CD4^+^ (PE), and anti-CD8^+^ (FITC) antibodies, and incubated at 4°C for 30 min in a dark place. Cells were washed with PBS thrice to remove unbound antibodies. The BD FACSAria III flow cytometry system (BD Biosciences, San Diego, CA, United States) was used to acquire the data. Finally, the population of CD4^+^ and CD8^+^ T lymphocytes was analyzed using FlowJo software (BD Biosciences) and expressed as percentages.

#### Quantification of Cytokines and Immunoglobulin by ELISA

The serum sample was obtained from whole blood by centrifugation for 10 min at 3000 rpm and kept at –70°C until use. The concentration of cytokines [interferon (IFN)-γ, interleukin (IL)-6, IL-2, tumor necrosis factor (TNF)-α, IL-1β, IL-4, and IL-10] and immunoglobulins (IgG) in the serum was determined according to the instructions of the ELISA kits. The results were expressed as pg/mL by generating a standard curve.

### Statistical Analysis

Data were expressed as the mean ± standard error of the mean. Comparisons among the groups were made using one-way analysis of variance, followed by Tukey’s multiple comparison test using GraphPad Prism software version 7 (GraphPad Software, Inc., San Diego, CA, United States). *p* < 0.05 was considered statistically significant.

## Results

### Scanning Electron Microscopy Analysis of hLR

**FIGURE 2:**
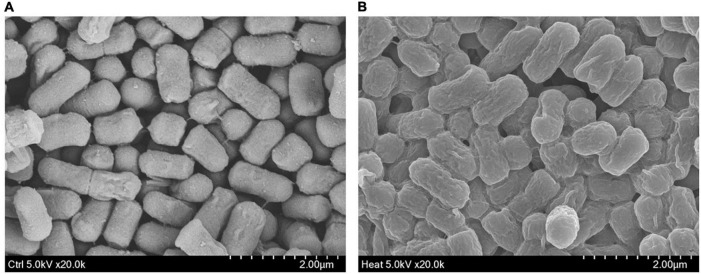
Morphology of control *L. reuteri* PSC102 **(A)** and hLR **(B)**.

Scanning electron microscopy was used to observe and compare the morphology of viable and heat-killed *L. reuteri* PSC102. Upon exposure of heat (80°C for 15 min), *L. reuteri* PSC102 was killed and caused a minor morphological alteration. SEM indicated that cell surface of heat-killed *L. reuteri* PSC102 appeared slightly rougher and uneven than live *L. reuteri* PSC102 (Figure 2).

### Analysis of the Phylogenetic Tree

**FIGURE 3:**
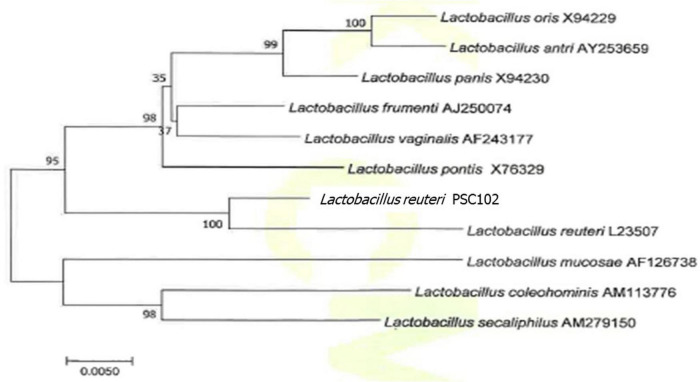
Phylogenetic tree of strain *L. reuteri* PSC102 based on the comparative analysis of the 16S rRNA sequence. Data were obtained from the NCBI-GenBank, and the phylogenetic tree was built with the neighbor-joining method using the Mega 5.0 program package. Scale bar, 0.0050 substitution per nucleotide.

The GenBank data homology search for the nucleotide sequence of the strain PSC102 16S rRNA gene at the National Center for Biotechnology Information (NCBI) showed that it belongs to *L. reuteri* with > 99% similarity. Strain *L. reuteri* PSC102 and *L. reuteri* L23507 belong to the same taxonomic group according to the phylogenetic tree based on the comparative analysis of the 16S rRNA gene (Figure 3). Moreover, the tree indicated that *L. reuteri* PSC102 was allocated to the species of *L. reuteri*.

### Effects of hLR on Body Weight

**FIGURE 4:**
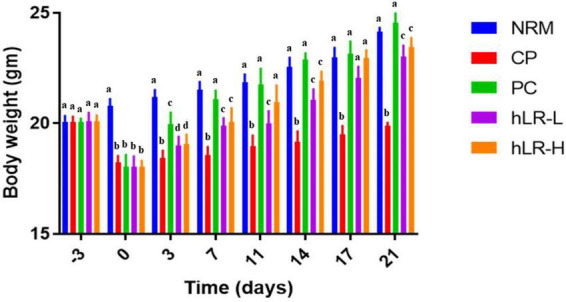
Effects of hLR on the body weight in CP-treated mice. Data are the mean ± SEM (*n* = 8). Values with different letters indicate a significant difference between the groups in the same time point (*p* < 0.05).

The body weight of mice is summarized in Figure 4. Before CP administration, the body weight did not differ among the different groups of mice. However, there was a significant weight loss due to injection with CP compared to NRM mice (*p* < 0.05). In the CP group, treatment with hLR-L, hLR-H, and levamisole (PC) showed a significant increase in body weight throughout the remaining experimental period compared to CP alone-treated group (*p* < 0.05).

### Effects of hLR on the Immune Organ Index

The thymus and spleen indices of the CP group were significantly decreased than the NRM group (*p* < 0.05; Table 2). However, the indices of both organs were significantly improved in the PC, hLR-L, and hLR-H groups compared to the CP group (*p* < 0.05). The hLR-H group showed a stronger effect on immune organ indices than the PC group (*p* < 0.05).

**TABLE 2:** Effects of hLR on the immune organ (thymus and spleen) index in mice.

Groups	Thymus index	Spleen index
NRM	2.65 ± 0.07^a^	4.66 ± 0.13^a^
CP	2.08 ± 0.10^b^	3.57 ± 0.05^b^
PC	2.88 ± 0.09^a^	4.49 ± 0.11^a^
hLR-L	2.72 ± 0.7^a^	4.15 ± 0.16^a^
hLR-H	3.29 ± 0.12^c^	5.56 ± 0.10^c^

*Data are the mean ± SEM (n = 8). Different letters indicate a significant difference between the groups (p < 0.05).*

### Effects of hLR on the Hematopoietic Functions in CP-Treated Mice

**FIGURE 5:**
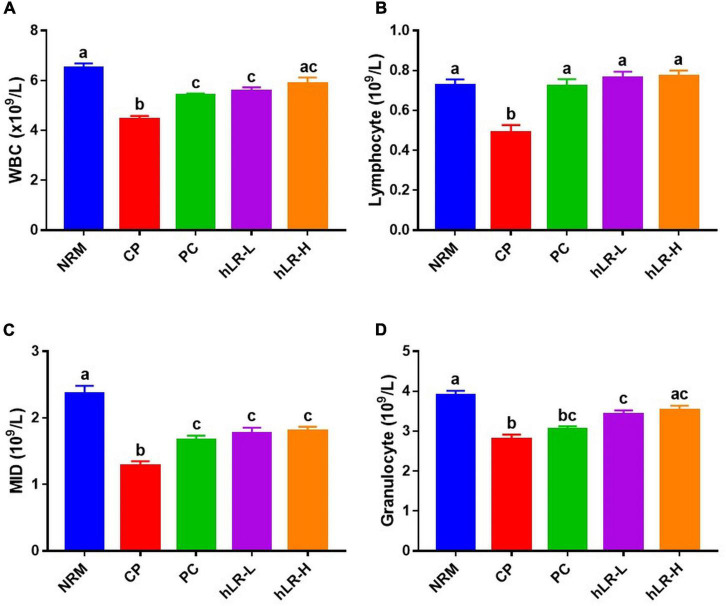
Effects of hLR on immune blood cell counts of CP-treated immunosuppressed mice. Number of WBCs **(A)**, lymphocytes **(B)**, MID **(C)**, and granulocytes **(D)**. Data are the mean ± SEM (*n* = 8). Different letters above the bars indicate a significant difference between the groups (*p* < 0.05).

To assess the protective effects of hLR on the myelosuppression produced by CP, WBCs, lymphocytes, MID, and granulocytes from peripheral blood were analyzed. WBCs, lymphocytes, MID, and granulocytes counts were significantly reduced (*p* < 0.05) in CP-treated mice compared to NRM mice (Figure 5). However, mice treated with hLR had significantly higher hematopoietic effects than mice in the CP group. A numeral increase in WBCs, lymphocytes, MID, and granulocytes counts was observed in both doses of hLR. This observation indicated that hLR could improve the myelosuppression induced by CP.

### Effects of hLR on the Splenocyte Proliferation of Immunosuppressed Mice

**FIGURE 6:**
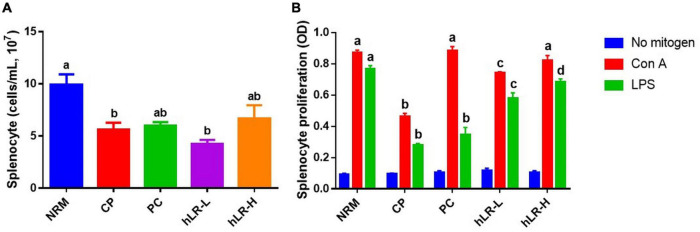
Effects of hLR on splenocyte proliferation. **(A)** Splenocyte counts in different groups. **(B)** Con A- and LPS-induced splenocyte proliferation. Data are the mean ± SEM (*n* = 4). Different letters above the bars indicate a significant difference between the groups (*p* < 0.05).

Splenocyte proliferation is one of the essential events in the activation event of cellular and humoral immunity (Sassi et al., 2017). Spleen cell counts in hLR-treated (hLR-H) mice were higher than in CP-treated mice (Figure 6A). Moreover, as shown in Figure 6B, CP treatment could decrease the proliferation of splenocytes compared to NRM mice. However, hLR treatment significantly enhanced the proliferation of splenic T lymphocytes compared to the CP-treated group (*p* < 0.05). This observation indicated that hLR could stimulate T-lymphocyte-specific proliferation capacity.

### Effects of hLR on the Expression of Thymic and Splenic T-Lymphocyte Subpopulations

**FIGURE 7:**
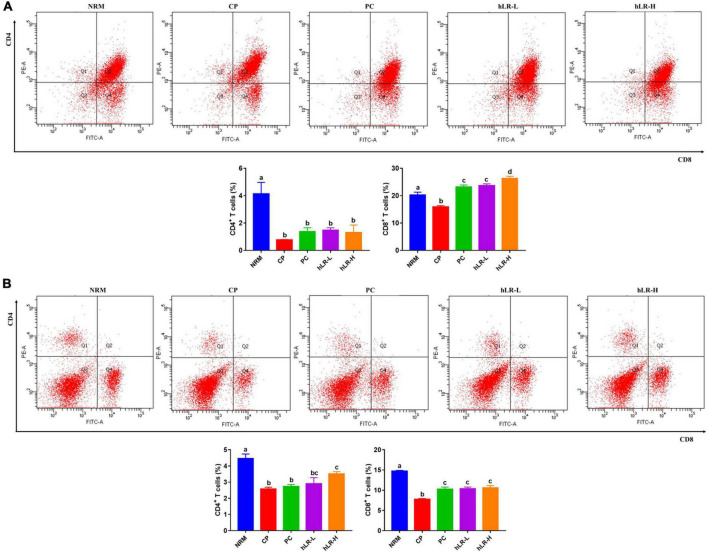
Effects of hLR on thymic and splenic T-lymphocyte subpopulations. **(A)** Thymic T-lymphocyte subpopulations (CD4^+^, region Q1; CD8^+^, region Q4). **(B)** Splenic T-lymphocyte subpopulations (CD4^+^, region Q1; CD8^+^, region Q4). Data are the mean ± SEM (*n* = 4). Different letters above the bars indicate a significant difference between the groups (*p* < 0.05).

To determine the effects of hLR on lymphocyte activities, thymic and splenic T-lymphocyte subsets were determined by flow cytometry. As shown in Figure 7A, the percentages of CD4^+^ and CD8^+^ T lymphocytes in the CP-treated group were significantly decreased compared to NRM (*p* < 0.05). However, CD4^+^ T-lymphocyte subpopulations in the thymus increased in mice treated with hLR compared to the CP-treated group, but the increase was non-significant. In contrast, the percentage of CD8^+^ T lymphocytes increased in the hLR-treated group, which was statistically significant (*p* < 0.05). In the spleen (Figure 7B), the expression of CD4^+^ T lymphocytes was significantly increased in mice treated with hLR-H, and the expression of CD8^+^ T lymphocytes was significantly increased in mice treated with both hLR-L and hLR-H than the CP-treated group (*p* < 0.05).

### Effects of hLR on Cytokine and Immunoglobulin Levels in the Serum of Mice

**FIGURE 8:**
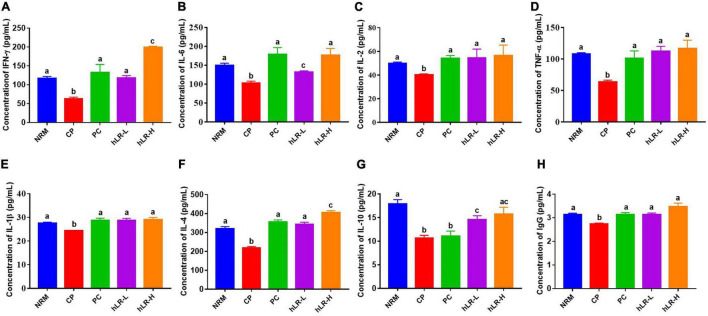
Effects of hLR on cytokines and immunoglobulin in the serum of mice. IFN-γ **(A)**, IL-6 **(B)**, IL-2 **(C)**, TNF-α **(D)**, IL-1β **(E)**, IL-4 **(F)**, IL-10 **(G)**, and IgG **(H)** concentrations. Data are the mean ± SEM (*n* = 3). Different letters above the bars indicate a significant difference between the groups (*p* < 0.05).

The cytokine and immunoglobulin levels are shown in Figure 8. IFN-γ, IL-2, and TNF-α are preferentially called Th1-type cytokines, whereas IL-4 and IL-10 are generally regarded as Th2-type cytokines (Saxena and Kaur, 2015). CP treatment caused a significant decrease in IFN-γ, IL-6, IL-2, TNF-α, IL-1β, IL-4, and IL-10 in the sera of the CP group compared to the NRM (*p* < 0.05). hLR treatment significantly prevented CP-treated decrease in both Th1- and Th2-type cytokines as well as other proinflammatory cytokines (IL-1β and IL-6). These cytokines significantly increased in both hLR groups, and the PC group compared to the CP group (*p* < 0.05). In immunoglobulin production, IgG production was significantly suppressed in the CP-treated group compared to NRM (*p* < 0.05). However, hLR administration was found to noticeably inhibit the decline in IgG concentrations.

## Discussion

Immunosuppression reduces the body’s capability to resist a particular infection due to damage to the immune system. It is an area of interest to discover immunomodulatory agents from probiotic bacteria to treat immunosuppressive diseases. Besides this, the attention to using inactivated probiotic bacteria as an immunostimulatory agent is increasing day by day. Recent investigations suggested that killed bacteria can produce bioactivities and are also believed to be safe (Campeotto et al., 2011; Taverniti and Guglielmetti, 2011). In our study, we killed *L. reuteri* PSC102 with optimal killing condition (80°C for 15 min). Heat inactivation neither damages the total cell integrity of *L. reuteri* PSC102 nor diminishes the capability to stimulate immune response in mice. A slight change in bacterial morphology can be happened during exposing to external stresses, such as high temperatures (Ou et al., 2011). Similarly, our SEM analysis demonstrated that after being exposed to high temperature, hLR retained its bacterial structure with a very slight rough and uneven cell surface. Various cell wall components of hLR could contribute to showing immunomodulatory effects. Many reports showed that lipoteichoic acid, teichoic acid, peptidoglycan, and β-glucan of bacteria, known as pathogen-associated molecular patterns (PAMPs), modulate the immune response by regulating cytokine production (Matsuguchi et al., 2003; Adams, 2010). Moreover, these components can also stimulate the proliferation of hematopoietic and lymphatic cells to boost immunity (Ohshima et al., 1991). Exopolysaccharide, an extracellular carbohydrate macromolecule secreted from probiotic bacteria has been characterized to possess pathogen protection and immunomodulating properties (Angelin and Kavitha, 2020). Moreover, bacterial exopolysaccharides are being studied extensively due to their biological activities and physicochemical features, as well as their prospective applications in industry, food, cosmetics, and medicine (Castro-Bravo et al., 2018; Angelin and Kavitha, 2020). Surface-layer proteins are present on the cell surface of lactobacilli, has been proved the capability of the probiotics to bind to immune cells such as dendritic cells to stimulate the T-regulatory phenotype and maintain the immune homeostasis (Konstantinov et al., 2008). Bacteriocins are antimicrobial tiny heat-stable peptides produced by lactic acid bacteria capable to prevent the growth of other pathogenic bacteria including enteric pathogens. Reuterin, a well-known antibacterial bioactive metabolite produced by *L. reuteri* has been shown to exert its activity against gut pathogenic microorganisms (Schaefer et al., 2010). Except bacteriocins and rueterin, the probiotic lactic acid bacteria contains a large number of anti-microbial components, such as diacetyl, lactic acid, and acidocin (Lukic et al., 2017).

Although the mechanism of action of probiotics on host immunity is not fully known. Several studies proposed some pathways to reveal the potential mechanism of action to regulate the immune system. The probiotic bacteria have the capability to interact with lymphocytes, macrophages, and dendritic cells to produce immune response which is triggered by pattern recognition receptors (PPRs) by binding with PAMPS (Gómez-Llorente et al., 2010). The well-known PPRs are toll-like receptors (TLRs). Among many other TLRs, TLR2 can recognize peptidoglycan, which is found in Gram-positive bacteria such as *Lactobacillus* genus. Previous studies showed that *L. casei* CRL431 interacts with epithelial cells via TLR2, and this interaction in gut associated immune cells encourages an increase in the number of TLR2 receptors to be involved in innate immune response (Vinderola et al., 2005). Moreover, *L. casei* CRL431 can activate TLR4 to induce proinflammatory mediators, leading to the recruitment of inflammatory cells, and initiate the immune response in spleen (Castillo et al., 2011). Furthermore, extracellular C-type lectin receptors (CLRs) and intracellular nucleotide binding oligomerization domain-containing protein (NOD)-like receptors (NLRs) have been found to send signals during interaction with bacteria and thereby contribute to the immune response (Lebeer et al., 2010). It has been demonstrated that immunobiotic *Lactobacillus* stains can induce appropriate NLR family pyrin domain containing protein (NLRP) 3 activation in swine gut-associated lymphoid tissue (GALT) by directly promoting NLRP3 expression through TLR and NOD-mediated signaling to maintain immune homeostasis (Tohno et al., 2011).

Cyclophosphamide, as an alkylating agent, has been widely applied for cancer treatment. Besides its significant clinical effects, it can produce cytotoxic effects by alkylating DNA, cross-linking proteins, impairing immunity, and interfering with the proliferation and differentiation of T and B cells. CP treatment can cause weight loss of immune organs and discrepancy of various leukocytes in the peripheral blood of mice, ultimately decreasing immune functions (Xu and Zhang, 2015). Meanwhile, it can reduce the expression of proinflammatory cytokines and thus suppress the cell-mediated and humoral immunity of the organism (Fan et al., 2013). Therefore, the immunosuppressed state of mice as an animal model was established by CP treatment to assess the immunostimulant effects of hLR in this study.

In this study, treatment of mice with CP (80 mg/kg, i.p.) notably decreased the body weight, immune organ index, hematopoietic functions, splenocyte proliferation, and expression of CD4^+^ and CD8^+^ T lymphocytes. Furthermore, the levels of cytokines IFN-γ, IL-6, IL-2, TNF-α, IL-1β, IL-4, and IL-10, and immunoglobulin IgG were reduced by CP. These experimental outcomes concurred with previous reports (Meng et al., 2018; Noh et al., 2019). The above-described results indicated that the immune functions of BALB/c mice significantly declined by CP, suggesting that the immunosuppressed model of mice was effectively produced.

The effects of hLR on the thymus and spleen indices were investigated first, as both are significant immune organs in the body and sites of immunological cell growth and proliferation. T lymphocytes grow, proliferate, differentiate, and mature in the thymus, whereas B lymphocytes mature in the bone marrow. After maturation, both T and B lymphocytes migrate to the different secondary lymphoid organs including spleen. As a result, the immune organ index is usually used to reveal immune organ growth and assess the immunoregulatory effects of probiotics (Li et al., 2011; Won et al., 2011). According to these findings, hLR treatment significantly increased the thymus and spleen indices compared to the CP group, indicating that hLR could resist the impact of immunosuppression on the development of vital immune organs.

The proliferation of lymphocytes in response to mitogens is usually used to determine the efficacy of immunomodulatory agents (Li et al., 2011); hence, splenic lymphocyte proliferation has been used to evaluate the effects of heat-killed probiotics on immune function. Heat-inactivated *Lactobacillus brevis* KCTC 12777BP can stimulate mitogen-induced splenocyte proliferation in a dose-dependent manner (Jeong et al., 2020). To examine cellular immunity, splenocytes of hLR-administered mice were isolated, and their proliferation was tested by treating them with mitogen Con A and LPS. Results showed that hLR treatment significantly increased the splenocyte proliferation of CP-induced immunosuppressed mice, providing the supporting evidence of improving cell-mediated and humoral immunity. Hence, hLR can play a crucial role in the initiation and modulation of non-specific immune responses.

Cycophosphamide-induced myelosuppression is a major issue for cancer patients undergoing clinical chemotherapy. Previous studies showed that CP administration significantly reduced hematopoiesis (Wang et al., 2012; Jang et al., 2013). hLR administration enhanced the activity of the hematopoietic system, suggesting that it can restore peripheral WBC, lymphocyte, MID, and granulocyte counts against the myelosuppression induced by CP.

T-helper (CD4^+^) and cytotoxic T (CD8^+^) lymphocytes are the most important immune cells regulating the immune system via the release of cytokines or direct cytotoxic effects (Jang et al., 2013). In accordance with the previous investigation, the expression of CD4^+^ and CD8^+^ T cells was markedly reduced in mice treated with CP than normal mice (Huyan et al., 2011; Włodarczyk et al., 2018). These findings suggested that hLR can prevent the decline in the proportion of CD4^+^ and CD8^+^ T-lymphocyte subpopulations. Based on these results, hLR possesses multiple positive impacts to augment cellular immunity in CP-treated mice. IgG is one of the major immunoglobulins that can boost humoral immunity to protect against different kinds of external infectious agents (Vidarsson et al., 2014). In this study, the result indicated that hLR could increase humoral immunity by stimulating IgG production.

Th1 and Th2 are two distinct types of T-lymphocyte subsets differentiated by activated CD4^+^ T cells. Activated Th cells are distributed into Th1 and Th2 after antigen recognition and secrete different cytokines. Normally, Th1 cells release IFN-γ, IL-2, and TNF-α, which mainly contribute to cell-mediated immune response (Letsch and Scheibenbogen, 2003; Li et al., 2013), whereas Th2 cells secrete IL-4 and IL-10, which mainly boost humoral immunity (Kidd, 2003). It is important to keep the functional kinetic equilibrium between Th1 and Th2 to maintain the host’s normal cell-mediated and humoral immune response. Probiotic *Lactobacillus* spp. show a substantial impact on the Th1/Th2 immune response (Torii et al., 2007). This investigation showed a significant increase in the levels of Th1-type cytokines (IFN-γ, IL-2, and TNF-α) in all hLR-treated groups compared to the CP group. Moreover, secretion of Th2-type cytokines (IL-4 and IL-10) was significantly elevated with hLR treatment in CP-treated immunosuppressed mice. Additionally, the non-Th1/Th2 cytokines (IL-1β and IL-6) also showed similar increasing trend in hLR treated mice. This observation indicated that hLR might maintain the normal immune function by inspiring the secretion of non-Th1/Th2 cytokines as well as regulating the balance between Th1 and Th2.

## Conclusion

This study demonstrated that oral administration of hLR improves immunity by stimulating the immune organ development, enhancing hematopoietic functions, increasing lymphocyte proliferation, improving the expression of T-lymphocyte subpopulations, and upregulating the levels of Th1/Th2 cytokines and non-Th1/Th2 cytokines, and immunoglobulins in CP-induced immunosuppressed mice. Therefore, these findings suggest that hLR can be used as an effective immunostimulating agent to ameliorate impaired immunity in humans and other animals. Nonetheless, the pharmacologically active components in hLR, as well as the signaling mechanisms involved in immunostimulation are also remained to be further elucidated.

## Data Availability Statement

The original contributions presented in this study are included in the article/supplementary material, further inquiries can be directed to the corresponding author.

## Ethics statement

The animal study was reviewed and approved by Laboratory Animal Care and Use Committee of Kyungpook National University (2020-103).

## Author contributions

S-CP, MA, and YQ: conception and design of the study. MA, E-BL, and BB: execution of the experiment. MA and YQ: acquisition and analysis of data. MA and S-CP: drafting of the manuscript. S-CP, KS, and S-KL: revision of the manuscript. S-CP and KS: supervision of the whole project. All authors have read and consented to the final version of the manuscript for publication.

## Conflict of Interest

The authors declare that the research was conducted in the absence of any commercial or financial relationships that could be construed as a potential conflict of interest.

## Publisher’s Note

All claims expressed in this article are solely those of the authors and do not necessarily represent those of their affiliated organizations, or those of the publisher, the editors and the reviewers. Any product that may be evaluated in this article, or claim that may be made by its manufacturer, is not guaranteed or endorsed by the publisher.

## Footnotes

1
https://www.ncbi.nlm.nih.gov/nuccore/2032707025
